# Successful Pregnancy in a Patient with Combined Deficiency of Factor V and Factor VIII

**DOI:** 10.1155/2014/343717

**Published:** 2014-05-04

**Authors:** Ahmed Ghassan El Adib, Farah Majdi, Mohamed Othmane Dilai, Hamid Asmouki, Ahlam Bassir, Karam Harou, Abderraouf Soumani, Said Younous, Lahoucine Mahmal

**Affiliations:** ^1^Department of Anesthesia and Critical Care, University Hospital Mohammed VI, Faculty of Medicine and Pharmacy, Cadi Ayyad University, Marrakesh, Morocco; ^2^Department of Obstetrics and Gynecology, University Hospital Mohammed VI, Faculty of Medicine and Pharmacy, Cadi Ayyad University, Marrakesh, Morocco; ^3^Department of Hematology and Oncology, University Hospital Mohammed VI, Faculty of Medicine and Pharmacy, Cadi Ayyad University, Marrakesh, Morocco

## Abstract

Inherited combined factor V and factor VIII deficiency (F5F8D) is autosomal recessive transmission disorder. Epistaxis, postsurgical bleeding, and menorrhagia are the most common symptoms. The risk of miscarriage and placental abruption is consequent. We report a case of successful pregnancy in a patient with F5F8D. 20-year-old woman, born of consanguineous parents, third gestate, first parity, two miscarriages, admitted for child birth of a spontaneous pregnancy estimated at 38 weeks and was diagnosed with F5F8D. At admission, patient was hemodynamically stable, with good obstetric conditions. The biologic results showed low levels of PT (52%), factor V (7%), and factor VIII (5%), and the activated partial thromboplastin time was prolonged (68,6%). Parturient was admitted in intensive care unit, maternal and fetal monitoring was performed. Fresh frozen plasma (FFP) and factor VIII concentrates were perfused at the induction of labor. Analgesia used fentanyl titration. The delivery gave birth to a newborn male, with Apgar 10/10 and 3000 g. The puerperium was simple without any important bleeding. Laboratory tests for the newborn were acceptable. Little literature is available on this subject and there are no guidelines available concerning pregnancy; we chose to prescribe a combination of factor VIII concentrate and FFP in pre-, per- and postpartum. The same protocol was successfully used in a patient before dental extraction and prostatectomy. Vaginal delivery is possible, as our case. Management by multidisciplinary team is recommended.

## 1. Introduction

Inherited combined factor V and factor VIII deficiency (F5F8D) is a rare occurrence.

147 cases have been reported worldwide until May 2006 [1, 2]. Most reported families are from the Middle East. Transmission is autosomal recessive, so parents of affected individuals are obligatory heterozygotes [3]. Recent studies identified mutations in two genes, lectin mannose binding protein 1 (*LMAN1*) and multiple coagulation factor deficiency 2 (*MCFD2*) as the cause of F5F8D [4]. Epistaxis, postsurgical bleeding, and menorrhagia are the most common symptoms [5]. Other types of bleeding can occur, including hemarthrosis and muscular hematomas [5]. In the last 20 years, there have been several case reports and case series documenting the profoundly increased risk of miscarriage and placental abruption resulting in foetal loss or premature delivery in women with bleeding disorders. No published data are available on the optimal management of women with F5F8D during pregnancy [6]. We report a case of successful pregnancy in a patient with F5F8D, to discuss the open issues of the management of the childbirth and the bleeding risk at delivery.

## 2. Case Report

The patient was 20 years old, born of consanguineous parents, third gestate, first parity, and two miscarriages. She was admitted for the child birth of a spontaneous pregnancy estimated at 38 weeks and 5 days.

Her past history revealed the concept of frequent bruising after falls and recurrent epistaxis, but no investigation had been made to coagulation. As part of her abortive disease, an assessment was made, and the laboratory tests showed low levels of prothrombin time (PT: 30%), FV (4%), and FVIII (5%), and the activated partial thromboplastin time was prolonged (APTT/111,1s), and normal levels of von Willebrand factor antigen (vWF : Ag) and ristocetin cofactor (Rcof) were found.

At the admission, the patient was hemodynamically good, outside of labor, not bleeding, and with good obstetric conditions. We had fetal wellbeing. The biologic results remained unchanged, with low levels of PT (52%), factor V (7%), and factor VIII (5%), and APTT was prolonged (68,6%).

The patient was admitted in intensive care unit, had both maternal and fetal monitoring, and received fresh frozen plasma (FFP) before, during, and after delivery; factor VIII concentrates were perfused at the induction of the labour. Analgesia used fentanyl titration. The delivery with episiotomy gave birth to a newborn male, with Apgar 10/10 and 3000 g. To prevent postpartum hemorrhage after delivery, the patient received oxytocin. The puerperium was simple. Laboratory tests for the newborn were acceptable.

## 3. Discussion

Combined FV and FVIII deficiency is a serious problem in obstetrics, because both the pregnancy and the delivery carry a high bleeding risk. There is little literature available on this subject and there are no guidelines available concerning pregnancy [5, 6]. To our knowledge, this is the first case reported in Morocco and the second worldwide [6].

F5F8D is a truly autosomal recessive disorder; heterozygotes have completely normal FV and FVIII levels, with an incidence of 1 per 1 million [7, 8]. It seems more common among Mediterranean populations especially in Jews and Iranians, where consanguineous marriages are frequent [2]. The deficiency results from mutations in either the* LMAN1* or* MCFD2* genes, which encode for proteins involved in the intracellular transport of FV and FVIII [9]. The synthesis of FV and FVIII within hepatocytes is normal, but intracellular trafficking and release into the circulation are impaired.

In F5F8D, our knowledge about the clinical manifestation so far is based on just few large series published in the literature; see Table 1 [5]. Patients with this disorder usually have mild to moderate bleeding symptoms and concomitantly low levels of FV and FVIII between 5% and 20% [9]. It was the case of our patient.

The FV level is likely to be the main determinant of bleeding risk at delivery, because FVIII levels increase during pregnancy but FV levels remain the same. FV and FVIII levels should be checked during the third trimester so that a delivery plan can be made [6]. Our patient results remained unchanged before and during the pregnancy. The latter does not seem to worsen this deficiency.

During labor, FFP at an initial dose of 15 to 20 mL/kg should be used to maintain FV levels at more than 15 IU/dL, and recombinant FVIII concentrate should be used to maintain FVIII levels at more than 50 IU/dL [6].

We chose to prescribe a combination of factor VIII concentrate and FFP before, during, and after delivery. The same protocol was successfully used in a patient before dental extraction [10] and prostatectomy [11], with this combined deficiency.

Vaginal delivery is possible at the termination of pregnancy, as in our case. Ueno et al. [6] reported a case of a successful Caesarean section for premature rupture of the membranes and an anomaly of rotation, while a trial of labor was indicated initially. If a Cesarean section is required, FV levels should be increased to more than 25 IU/dL and factor replacement should continue until wound healing has occurred [12–14].

Pregnancy and childbirth present a major challenge to women with inherited bleeding disorders. All women should be managed by a multidisciplinary team in a center where the expertise, laboratory support, and factor treatment required to provide care for these patients are available at all times. Additional reports are needed for establishing optimal guidelines for hemorrhagic, invasive, and surgical procedures in individuals with combined factors V and VIII deficiency.

## Figures and Tables

**Table 1:** Factor V and factor VIII deficiency's series published in the literature.

	Viswabandya et al. [5] (*n* = 37)	Peyvandi et al. (*n* = 27)	Shetty et al. (*n* = 9)	Seligsohn et al. (*n* = 14)	Mansouritorghabeh et al. [10] (*n* = 19)
Easy bruisability	30.00%		44.00%	29.00%	
Prolonged bleeding after injury/surgical procedure	62.00%	77.00%	77.00%	85.00%	73.00%
Menorrhagia	66.00%	58.00%	75.00%	100.00%	40.00%
Gum bleeding	49.00%		44.00%	64.00%	
Haemarthrosis	13.00%	25.00%	0.00%	0.00%	36.00%
Epistaxis	19.00%	77.00%		57.00%	48.00%
FV, *C* (%)	12.5%	11.00%	6.60%	17.00%	9.28%
FVIII, *C* (%)	8.80%	13.00%	1.60%	19.00%	12.63%

## References

[1] Farah RA, de Moerloose P, Bouchardy I (2006). Combined factor V-factor VIII deficiency (F5F8D): compound heterozygosity for two novel truncating mutations in LMAN1 in a consanguineous patient. *Thrombosis and Haemostasis*.

[2] Pathare A, Al Kindi S, Al Abri Q, Al Haddabi H, Gravell D, Dennison D (2004). Spectrum of inherited bleeding disorder from sultanate of Oman. *Haemophilia*.

[3] Faioni EM, Fontana G, Carpani G (2003). Review of clinical, biochemical and genetic aspects of combined factor V and factor VIII deficiency, and report of a new affected family. *Thrombosis Research*.

[4] Zhang B (2009). Recent developments in the understanding of the combined deficiency of FV and FVIII. *British Journal of Haematology*.

[5] Viswabandya A, Baidya S, Nair SC (2010). Clinical manifestations of combined factor V and VIII deficiency: a series of 37 cases from a single center in India. *American Journal of Hematology*.

[6] Ueno H, Asami M, Yoneda R (1991). Management of cesarean section under replacement therapy with factor VIII concentrates in a pregnant case with congenital combined deficiency of factor V and factor VIII. *Rinsho Ketsueki*.

[7] Mannucci PM, Duga S, Peyvandi F (2004). Recessively inherited coagulation disorders. *Blood*.

[8] Giddings JC, Seligsohn U, Bloom AL (1977). Immunological studies in combined factor V and factor VIII deficiency. *British Journal of Haematology*.

[9] Zhang B, McGee B, Yamaoka JS (2006). Combined deficiency of factor V and factor VIII is due to mutations in either LMAN1 or MCFD2. *Blood*.

[10] Mansouritorghabeh H, Rezaieyazdi Z, Bagheri M (2009). Successful use of factor viii concentrate and fresh frozen plasma for four dental extractions in an individual with combined factor v and VIII deficiency. *Transfusion Medicine and Hemotherapy*.

[11] Bauduer F, Guichandut JP, Ducout L (2004). Successful use of fresh frozen plasma and desmopressin for transurethral prostatectomy in a French Basque with combined factors V +VIII deficiency. *Journal of Thrombosis and Haemostasis*.

[12] Bolton-Maggs PHB, Perry DJ, Chalmers EA (2004). The rare coagulation disorders—review with guidelines for management from the United Kingdom Haemophilia Centre Doctors’ Organisation. *Haemophilia*.

[13] Kadir R, Chi C, Bolton-Maggs P (2009). Pregnancy and rare bleeding disorders. *Haemophilia*.

[14] Spreafico M, Peyvandi F (2008). Combined FV and FVIII deficiency. *Haemophilia*.

